# The Bioactive Peptide SL-13R Expands Human Umbilical Cord Blood Hematopoietic Stem and Progenitor Cells In Vitro

**DOI:** 10.3390/molecules26071995

**Published:** 2021-04-01

**Authors:** Takenobu Nii, Katsuhiro Konno, Masaki Matsumoto, Kanit Bhukhai, Suparerk Borwornpinyo, Kazuhiro Sakai, Suradej Hongeng, Daisuke Sugiyama

**Affiliations:** 1 Incubation Center for Advanced Medical Science, Kyushu University, 3-1-1 Maidashi, Higashiku, Fukuoka 812-8582, Japan; niitake@jsd.med.kyushu-u.ac.jp (T.N.); kkonno@med.kyushu-u.ac.jp (K.K.); 2 Department of Research and Development of Next Generation Medicine, Faculty of Medical Sciences, Kyushu University, 3-1-1 Maidashi, Higashiku, Fukuoka 812-8582, Japan; 3 Department of Stem Cell Biology and Medicine, Graduate School of Medical Sciences, Kyushu University, 3-1-1 Maidashi, Higashiku, Fukuoka 812-8582, Japan; 4 Division of Cell Biology, Department of Molecular and Cellular Biology, Medical Institute of Bioregulation, Kyushu University, 3-1-1 Maidashi, Higashiku, Fukuoka 812-8582, Japan; masakim@bioreg.kyushu-u.ac.jp; 5 Department of Physiology, Faculty of Science, Mahidol University, 272 Rama VI Road, Ratchatewi, Bangkok 10400, Thailand; kanitscmu@gmail.com; 6 Department of Biotechnology, Faculty of Science, Mahidol University, 272 Rama VI Road, Ratchatewi, Bangkok 10400, Thailand; bsuparerk@gmail.com; 7 Angel Hospital, 1-11-1 Tomoda, Yahatanishiku, Kitakyushu 807-0828, Japan; spcp8zf9@river.ocn.ne.jp; 8 Department of Pediatrics, Faculty of Medicine Ramathibodi Hospital, Mahidol University, 270 Rama VI Road, Ratchatewi, Bangkok 10400, Thailand; suradej.hon@mahidol.ac.th; 9 Translational Research Center in Hiroshima University, 1-2-3 Kasumi, Minami-Ku, Hiroshima 734-8551, Japan

**Keywords:** umbilical cord blood, hematopoietic stem/progenitor cell, peptide, cell culture

## Abstract

Hematopoietic stem and progenitor cell (HSPC) transplantation is a curative treatment of hematological disorders that has been utilized for several decades. Although umbilical cord blood (UCB) is a promising source of HSPCs, the low dose of HSPCs in these preparations limits their use, prompting need for ex vivo HSPC expansion. To establish a more efficient method to expand UCB HSPCs, we developed the bioactive peptide named SL-13R and cultured UCB HSPCs (CD34+ cells) with SL-13R in animal component-free medium containing a cytokine cocktail. Following 9 days of culture with SL-13R, the numbers of total cells, CD34+, CD38− cells, and hematopoietic stem cell (HSC)-enriched cells were significantly increased relative to control. Transplantation of cells cultured with SL-13R into immunodeficient NOD/Shi-scid/IL-2Rγ knockout mice confirmed that they possess long-term reconstitution and self-renewal ability. AHNAK, ANXA2, and PLEC all interact with SL-13R. Knockdown of these genes in UCB CD34+ cells resulted in reduced numbers of hematopoietic colonies relative to SL-13R-treated and non-knockdown controls. In summary, we have identified a novel bioactive peptide SL-13R promoting expansion of UCB CD34+ cells with long-term reconstitution and self-renewal ability, suggesting its clinical use in the future.

## 1. Introduction

Hematopoietic stem cells (HSCs) are somatic stem cells that give rise to multiple types of mature and functional blood cells. HSCs have been applied to HSC transplantation for more than 50 years as curative treatment for hematological disorders such as anemia and leukemia [1]. Currently, bone marrow (BM), peripheral blood (PB) and umbilical cord blood (UCB) serve as cell sources for HSC transplantation. Among these, in the last decades UCB has attracted increasing attention because UCB have several advantages: their collection is non-invasive and safe for the donor, permissive of HLA mismatch and lower risk of graft-versus-host disease compared to BM or PB-derived CD34+ hematopoietic stem and progenitor cells (HSPCs) [2,3]. Nonetheless, utilization of UCB is limited as doses of HSPCs are lower in the UCB graft, which associated with inefficient engraftment and treatment-related mortality [4,5].

To overcome this limitation, investigators have established methods to expand UCB HSPCs ex vivo. Given that hematopoietic cytokines regulate HSPC function, cells are normally incubated with a cytokine cocktail during the expansion period [6,7]. Small molecules, such as nicotinamide, StemRegenin 1 (SR-1), and UM171 promote expansion of UCB HSPCs without loss of repopulating ability [8,9,10]. HSPCs expanded by these small molecules have been tested in a phase 1–2 trial and showed safety, feasibility, and efficacy [11,12,13,14].

During fetal development, HSCs emerge from hemogenic endothelium in the aorta-gonads-mesonephros (AGM) region [15,16,17], and then expand in liver [18]. Delta-like homologue 1 (DLK1) is expressed on hepatoblasts, which serve as an HSC niche and function in HSC maintenance and expansion in liver during fetal development [19,20,21]. Overexpression of DLK1 in hematopoietic cell inhibits differentiation [22]. The extracellular domain of DLK1 can be cleaved and give rise to a soluble fragment. This soluble DLK1 has potential to stimulate angiogenesis [23] and inhibit differentiation of adipocyte [24]. Given these findings, we hypothesized that peptide derived from the extracellular domain of DLK1 can expand HSPCs without loss of stemness.

Here, we report the development of a bioactive peptide named SL-13R, the design of which was originally derived from sequence of the extracellular domain of DLK1 protein. When UCB HSPCs were cultured with SL-13R in combination with hematopoietic cytokines under animal component free conditions. SL-13R enhanced ex vivo expansion of UCB HSPCs without loss of long-term reconstitution ability. We also identified proteins that interact with SL-13R in UCB HSPCs and analyzed their potential effects on colony formation of UCB HSPCs. Overall, our work suggests an ex vivo culture method to expand UCB-derived HSPCs useful for transplantation therapy.

## 2. Results

### 2.1. SL-13R Enhances Expansion of UCB-Derived Hematopoietic Cells

**Figure 1:**
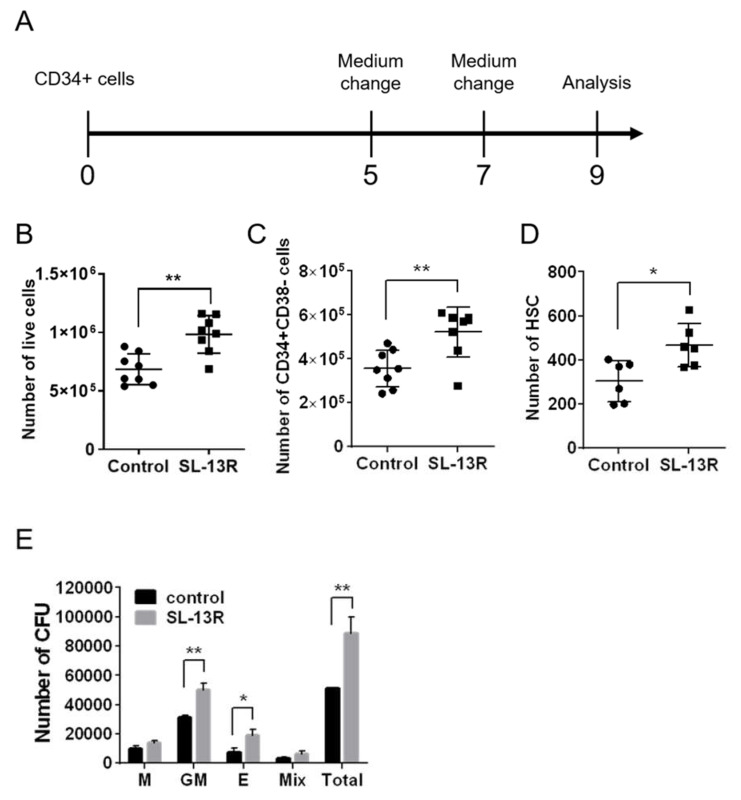
Ex vivo expansion of human UCB HSPCs by SL-13R peptide. (**A**) Human UCB CD34+ cells were cultured with or without SL-13R peptide (10 μg/mL) for 9 days and analyzed (**B**) The number of live cells with or without SL-13R (control: *n =* 8, SL-13R: *n =* 8). (**C**) The number of CD34+ CD38- cells with or without SL-13R (control: *n =* 8, SL-13R: *n =* 8). (**D**) The number of HSCs (CD34+, CD38−, CD45RA−, CD90+, CD49f+ cells) with or without SL-13R (control: *n =* 6, SL-13R: *n =* 6). (**E**) The number of colony forming unit (CFU) with or without SL-13R (control: *n =* 3, SL-13R: *n =* 3). The con-trol was PBS treatment. Student t test was used to test intergroup differences. * *p* < 0.05, ** *p* < 0.01.

To evaluate the effects of the bioactive peptide, SL-13R, on proliferation, we cultured UCB HSPC with or without SL-13R using a previously described protocol [7] with modifications under xeno-free conditions and then analyzed the number of live cells and HSPC population (Figure 1A). After 9 days of culture, we observed a 1.4-fold increase (*p* = 0.0012, *n* = 8) in the total number of living cells cultured with SL-13R compared to the cells cultured without SL-13R (Figure 1B). Flow cytometry was used to evaluate the number of CD34+, CD38– cells and HSCs (CD34+, CD38−, CD90+, CD45RA−, CD49f+) [25] on DAY9 and observed a significantly greater number of CD34+, CD38– cells and HSCs in SL-13R-treated cells compared to control (CD34+, CD38– cells: 1.5-fold higher, *p* = 0.0049, *n* = 8; HSCs: 1.5-fold higher, *p* = 0.014, *n* = 6) (Figure 1C,D). There was no significant difference in percentage of live cells (Figure S1). These effects were also observed with peripheral blood CD34+ cells cultured for 9 days with SL-13R (Figure S2A: *p* = 0.0048, Figure S2B: *p* = 0.60). To analyze the hematopoietic potential of cells cultured with SL-13R, we performed colony forming unit (CFU) assay. As shown in Figure 1E, SL-13R-treated cells yielded a significantly greater number of granulocyte and macrophage (GM), erythroid, and total colony forming unit compared to control.

### 2.2. Ex Vivo Expanded CD34+ Cells Treated with SL-13R Possess Long-Term Reconstitution and Self-Renewal Ability

**Figure 2:**
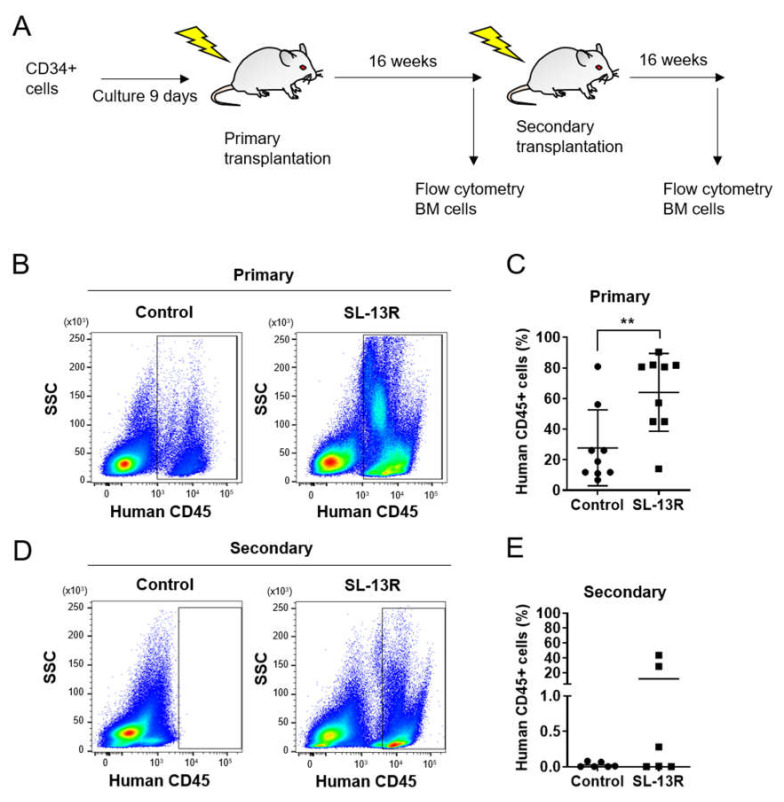
SL-13R-treated UCB CD34+ cells possess long-term reconstitution ability. (**A**) Experimental design of reconstitution assay. NOG mice were irradiated at 2.5 Gy and used as recipients for transplantation. Sixteen weeks after transplantation, bone marrow cells were harvested from 2 femurs and 2 tibias of recipient and expression of human-CD45 was assessed by flow cytometry. (**B**) Representative flow cytometric images of BM MNCs from recipient mice at primary transplantation. (**C**) The percentage of human-CD45+ cells at primary transplantation. *n =* 9 (9 UCB donors) for both control and SL-13R. ** *p* < 0.01. (**D**) Representative flow cytometric images of BM MNCs from recipient mice at secondary trans-plantation. (**E**) The percentage of human-CD45+ cells. *n =* 5 for control (one mouse was dead after trans-plantation), and *n =* 6 for SL-13R peptide (6 UCB donors).

To determine whether the cells expanded in the presence of SL-13R maintain long-term hematopoietic reconstitution ability, we transplanted UCB HSPC, cultured in the presence or absence of SL-13R, into immunodeficient NOD/Shi-scid/IL-2Rγ knockout (NOG) mice (Figure 2A). Sixteen weeks post-transplantation, we assessed the frequency of human CD45+ cells in the recipient mouse bone marrow. Percentages of human CD45+ cells in BM were 64 ± 25.35% in SL-13R-treated conditions and 27.74 ± 24.80% in controls (*n =* 9, *p* = 0.0074) (Figure 2B,C). To investigate the self-renewal ability, secondary transplantations were performed. None of the secondary transplant recipient mice showed reconstitution whereas 3 of 6 animals transplanted with human CD45+ cells under SL-13R-treated conditions showed reconstitution (12.10 ± 19.22%, *n =* 6, *p* = 0.15) (Figure 2D,E). These data indicate that SL-13R peptide expands number of HSCs without losing their long-term reconstituting and self-renewal ability.

### 2.3. SL-13R Induced Subset of CD34+ Cells Expansion as Potently as SR-1 and UM171

**Figure 3:**
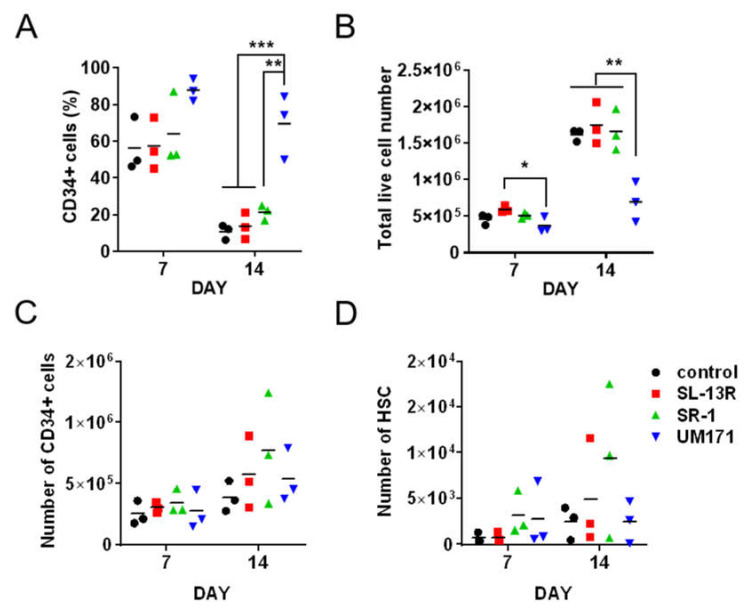

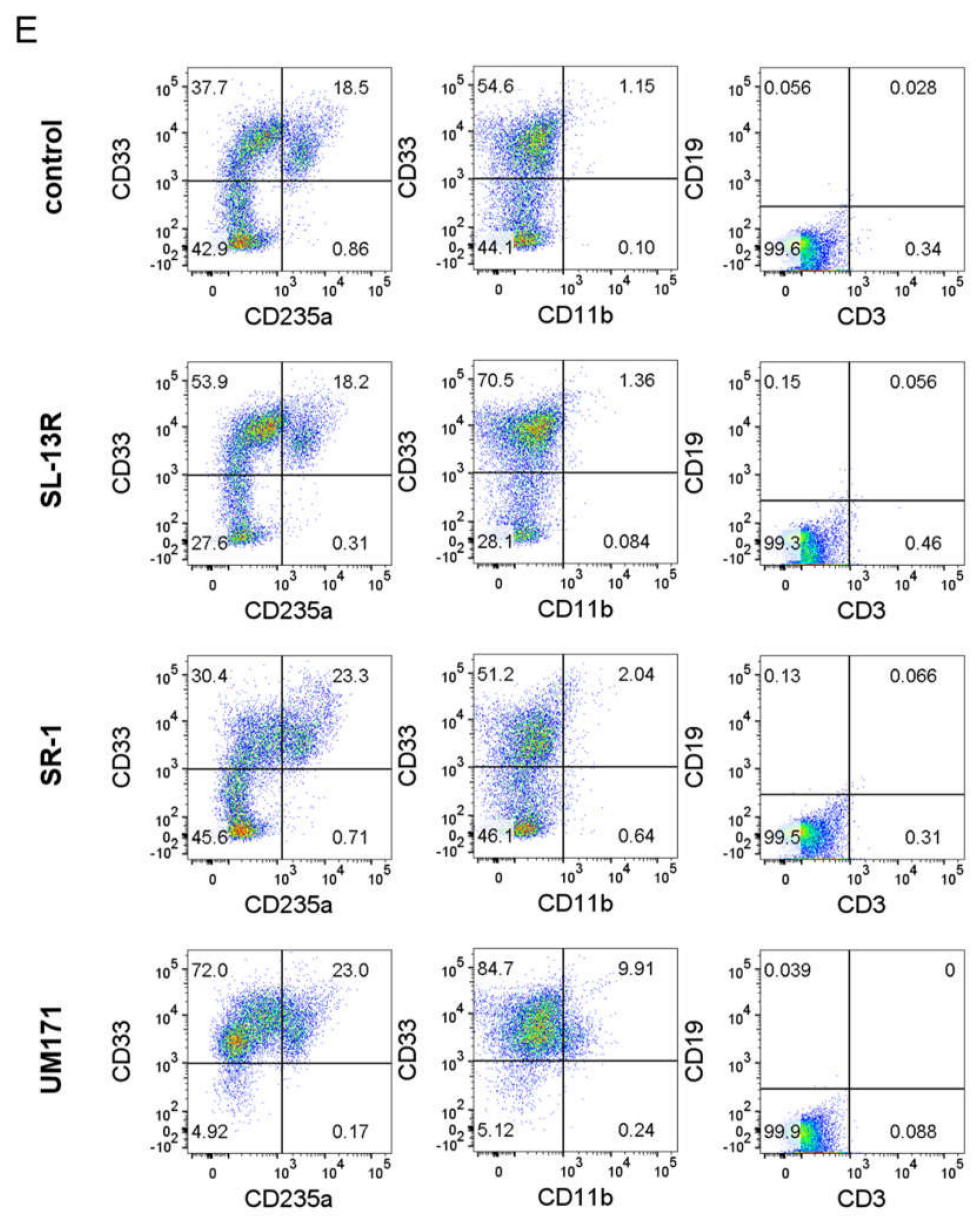
SL-13R expands HSPC number as potently as SR-1 and UM171. (**A**) The percentage of CD34+ cells cultured in the presence of SL-13R, SR-1, or UM171 for 7 and 14 days. (**B**) The number of live cells cultured in the presence of SL-13R, SR-1, or UM171 for 7 and 14 days. (**C**) The number of CD34+ cells cultured in the presence of SL-13R, SR-1, or UM171 for 7 and 14 days. (**D**) The number of HSCs (CD34 + CD38 − CD45RA − CD90 + CD49f + cells) in the presence of SL-13R, SR-1, or UM171 for 7 and 14 days. (**A**–**D**) *n =* 3; 3 UCB donors. The control was PBS treatment. One-way analysis of variance followed by Tukey’s post hoc test was used to test intergroup differences. ** *p* < 0.01, *** *p* < 0.005. (**E**) Percentages of CD33, CD235a, CD11b, CD19, and CD3 positive cells cultured in the presence of SL-13R, SR-1, or UM171 for 9 days.

We next compared the effects of SL-13R peptide with SR-1 [9] and UM171 [8], small molecules reported to expand UCB CD34+ cells. UM171 maintained CD34+ cells in vitro at a higher frequency compared to SL-13R and SR-1(Figure 3A) but did not increase live cell number (Figure 3B). In contrast, SL-13R increased live cell number (Figure 1B and Figure 3B). SR-1 exhibited a tendency to expand CD34+ cells and HSCs most efficiently however this trend was not statistically significant (Figure 3C,D); ( CD34+; DAY7 *p* = 0.74; DAY14 *p* = 0.51; HSC; DAY7 *p* = 0.40; DAY14 *p* = 0.40). To examine the effects of SL-13R, SR-1, and UM171 on expression of differentiation markers, we analyzed differentiation markers, such as CD33 (myeloid), CD11b (myeloid), CD235a (erythroid), CD3 (T cell), CD19 (B cell) by flow cytometry. SL-13R and UM171 increased the percentage of CD33+ cells (Figure 3E). SR-1 and UM171 increased the percentage of CD235a+ cells (Figure 3C), while UM171 increased the frequency of CD11b+ cells (Figure 3E). These data indicate that SL-13R induced subset of CD34+ cells expansion as potently as SR-1 and UM171.

### 2.4. Identification of Proteins Binding to SL-13R Peptide

To understand the mechanisms of SL-13R, we performed cDNA microarray analysis of CD34+ cells cultured with or without SL-13R for 2 days. We selected 345 up-regulated genes and 394 down-regulated genes (fold change > ±2 and Z-score > ±2) and analyzed them by Ingenuity Pathway Analysis (IPA) software. We found that several pathways regulating cell cycle and proliferation, such as protein kinase A, PI3K/AKT, AMPK signaling were upregulated by SL-13R treatment (Table 1). Indeed, SL-13R regulated cell cycle and cell proliferation related genes (Table S1 and Table S2) and increased BrdU+ cells during in vitro culture of mobilized PB CD34+ cells (Figure S3). These findings indicated that SL-13R expands HSPCs by regulating cell cycle and proliferation pathways and supports our conclusion that SL-13R increases the number of HSPCs in culture.

**Figure 4:**
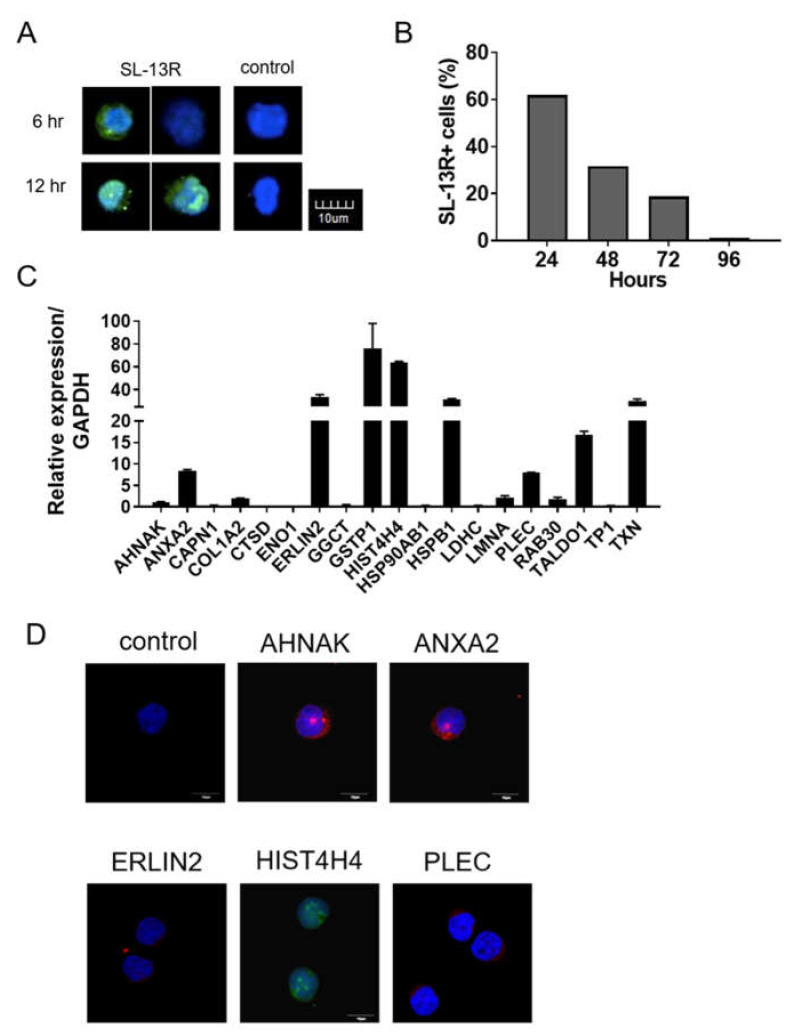
SL-13R is taken up by CD34+ cells. (**A**) Immunocytochemistry image of CD34+ cells cultured with or without biotin-conjugated SL-13R for 6 or 12 h. Biotin-conjugated SL-13R was detected using AlexaFluor 488-conjugated streptavidin (green), and TOTO-3 iodide (blue) was used for served as a nuclear staining. Scale bar = 10 μm. (**B**) Flow cytometric analysis of CD34+ cells cultured with biotin-conjugated SL-13R for 24 h and then washed out bio-tin-conjugated SL-13R from medium and cultured for 72 h (total 96 h) and analyzed every 24 h. Bio-tin-conjugated SL-13R was detected using APC-conjugated streptavidin. *n =* 1 (**C**) Relative mRNA expression of candidate genes for molecular partners of SL-13R in CD34+ cells. *n =* 3 (**D**) Immunocytochemical analysis of candidate gene in CD34+ cells.

As shown in Figure 4A, six hr post-addition of SL-13R, we detected SL-13R in the cytoplasm of cells. To understand the stability of SL-13R in cytoplasm, we washed out SL-13R from the culture media after 24 h and cultured for 72 h (total 96 h). The proportion of cells containing the SL-13R gradually decreased and the SL-13R was undetectable at 96 h after addition (Figure 4B). To further understand how SL-13R contributes to HSPC expansion, we investigated potential molecular partners of SL-13R by immunoprecipitation and detection with high performance liquid chromatography-tandem mass spectrometry (LC-MS/MS). We screened 20 molecules (peptides) by MASCOT search (shown in Table S3) and assessed whether corresponding transcripts were expressed in UCB and mobilized PB CD34+ cells (Figure 4C and Figure S4). Among them, we selected *AHNAK, ANXA2*, *ERLIN2*, *HIST4H4*, and *PLEC* and detected the protein expression in UCB CD34+ cells (Figure 4D).

**Figure 5:**
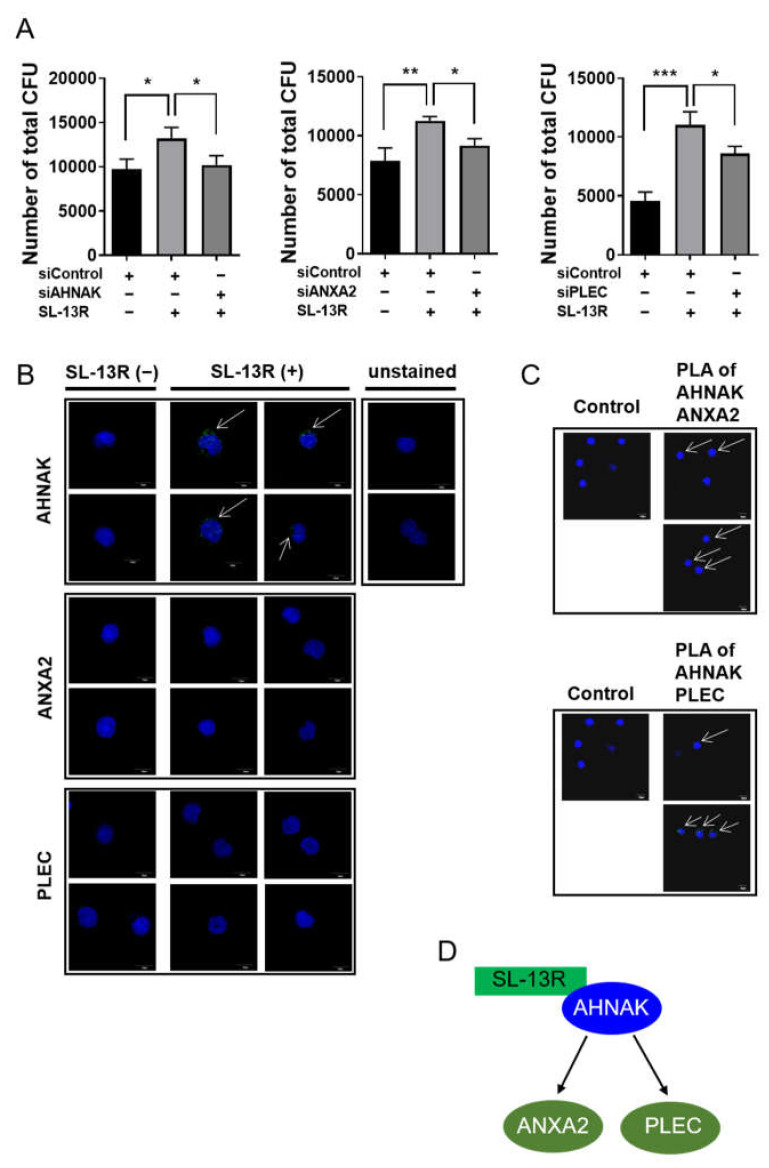
SL-13R directly binds with AHNAK. (**A**) Number of total CFU. After siRNA transfection, the cells were cultured with or without SL-13R for 3 days and performed CFU assay (*n =* 3). One-way analysis of variance followed by Tukey’s post hoc test was used to test intergroup differences. * *p* < 0.05, ** *p* < 0.01, *** *p* < 0.005. (**B**) Proximity ligation assay of biotin-conjugated SL-13R and AHNAK, ANXA2, or PLEC. (**C**) Proximity ligation assay of AHNAK and ANXA2 or PLEC. Control is negative control of no primary antibody. (**D**) Schematic diagram of proximity ligation assay result.

To determine whether these genes function in UCB HSPC in the presence of SL-13R, we conducted loss-of-function analysis by siRNA transfection. Three days after transfection of respective siRNAs, we collected cells and subjected them to CFU assays. The ability of SL-13R to increase CFU number was diminished by knockdown of AHNAK, AXNA2, and PLEC (Figure 5A and Figures S5 and S6). To further investigate peptide-protein interaction of SL-13R and AHNAK, AXNA2, or PLEC, we performed a proximity ligation assay. As shown in Figure 5B, we detected positive signal in proximity ligation assay of AHNAK, but not ANXA2 and PLEC. We also analyzed protein–protein interaction of AHNAK and ANXA2 or PLEC. We found positive signal in proximity ligation assay of AHNAK-ANXA2 and AHNAK-PLEC (Figure 5C). These results suggest that SL-13R directly interacts with AHNAK and indirectly interacts with ANXA2 and PLEC to expand HSPC (Figure 5D).

## 3. Discussion

In this study, we developed an ex vivo expansion method of CB CD34+ cells using the bioactive peptide SL-13R under animal component-free conditions. SL-13R expands CB CD34+ cells while maintaining long-term reconstitution ability in immunodeficient mice. The ability of SL-13R to expand CB CD34+ cells was as potent as those reported by others for the small molecules, such as SR-1 and UM171 in our culture system. Collectively, we propose a novel ex vivo CB CD34+ cells expansion method using bioactive peptide SL-13R.

SL-13R induced CB CD34+ cells expansion as effectively as SR-1 and UM171 in our study. SR-1 inhibits aryl hydrocarbon receptor signaling and maintains CD34+ cells in vitro [9,26]. UM171 was identified by screening of small molecules to expand mobilized peripheral blood CD34+CD45RA− cells and efficiently retains the CD34+CD45RA− cells [8] and regulates epigenetic state of HSPCs via CLR3-KBTBD4 complex and lysine-specific demethylase 1A [27,28]. SL-13R did not affect the proportion of CD34+ cells but increased the number of CD34+ cells without loss of reconstitution activity. Indeed, the genes related with cell cycle and proliferation are up-regulated in two days culture of CD34+ cells with SL-13R.

AHNAK, ANXA2 and PLEC as functional binding partners of SL-13R in UCB CD34+ cells. The functions of AHNAK, ANXA2, and PLEC in UCB CD34+ cells are poorly understood. AHNAK is a scaffold protein implicated in diverse biological processes, such as blood–brain barrier formation, cell architecture and migration, regulation of cardiac calcium channels and muscle membrane repair [29]. AHNAK is up-regulated in HSPCs of zebrafish, mouse, and human, and might be involved in the regulatory network of HSPC self-renewal and/or differentiation [30]. ANXA2 is secreted protein and is involved in diverse cellular processes, such as cell motility, endocytosis and cell matrix interactions [31] and is a known modulator of the HSC niche being expressed by endosteal osteoblasts [32]. Recently, Jin et al. show that Ahnak is a major binding partner of the p11/Anxa2 complex and regulates calcium channel in neurons [33]. Plectin is an intermediate filament-associated protein and acts as a cytoskeletal crosslinker and signaling scaffold [34]. Protein–protein interactions between AHNAK and PLEC are reported in lens fiber cells [35]. PLEC and AHNAK are detected as interaction partners of Formin-like 1 (FMNL1) in hematopoietic cells and are involved in calcium-dependent membrane processes [36]. These finding support our findings which suggest that SL-13R-AHNAK-ANXA2 or SL-13R-AHNAK-PLEC complexes may form in UCB CD34+ cells. Knockdown of AHNAK, ANXA2, and PLEC decreased the effect of SL-13R in CFU assay. We suggest that SL-13R and AHNAK, ANXA2, or PLEC are involved in UCB CD34+ cells expansion. A better mechanistic understanding of how SL-13R and AHNAK, ANXA2, and PLEC induces UCB CD34+ cells expansion has yet to be developed in future studies.

In summary, we report the identification of a novel bioactive peptide, SL-13R, that promotes efficient expansion of UCB CD34+ cells with long-term reconstitution ability. Our results show that use of this peptide may substantially improve use of UCB as a source of UCB CD34+ cells and broaden their clinical application. Further studies to elucidate mechanisms by which this peptide promotes UCB CD34+ cells expansion could suggest methods to optimize its use.

## 4. Materials and Methods

### 4.1. Cell Culture

Human UCB CD34+ cells (Riken BioResource Center, Ibaraki, Japan) were thawed using a ThawSTAR automated cell thawing system (Biocision, San Rafael, CA, USA). Human UCB were also provided from Dr. Kazuhiro Sakai (Angel Hospital, 1-11-1, Tomoda, Yahatanishi-Ku, Kitakyushu, Fukuoka, Japan). UCB mononucleated cells (MNCs) were separated by Lymphoprep (Nyegaard A/S, Oslo, Norway). CD34+ cells were sorted from UCB MNCs by EasySep Human CD34 Positive Selection Kit II (STEMCELL Technologies Inc., Vancouver, BC, Canada). Mobilized peripheral blood CD34+ cells were obtained from leftover specimens of leukapheresis at Ramathibodi Hospital, Mahidol University. CD34+ cells were cultured in StemSpan-ACF medium (STEMCELL Technologies) supplemented with penicillin and streptomycin (Wako, Tokyo, Japan), 50 ng/mL human stem cell factor (SCF), 10 ng/mL thrombopoietin (TPO), 20 ng/mL FMS-like tyrosine kinase 3 ligand (FLT3L), 20 ng/mL interleukin 6 (IL-6), and 20 ng/mL soluble IL-6 receptor α (sIL-6Rα) (all from PeproTech, Rocky Hill, NJ, USA) with or without 10 μg/mL SL-13R peptide (Science Lustre Ltd., Fukuoka, Japan). The cells were incubated in 5% CO2 at 37℃ On DAY5 and DAY7, cells were split into 2 wells and supplemented with fresh medium. On DAY9, cells were collected and analyzed. The number of live cells was counted using trypan blue on a hemocytometer. The number of CD34+, CD38− cells and HSCs was determined by multiplying the number of live cells by the percentage of CD34+, CD38− cells and HSCs, respectively. This study has been approved by Kyushu University Institutional Review Board for Clinical Research and Institutional Review Boards in Mahidol University.

### 4.2. Flow Cytometry

Cultured cells were blocked with staining buffer (2% fetal bovine serum (FBS) phosphate-buffered saline (PBS)), and then incubated with the antibodies listed in Table S4. The cells were detected on a FACSAria cell sorter (BD Biosciences, San Jose, CA, USA), followed by data analysis using FlowJo software (BD Biosciences, San Jose, CA, USA).

### 4.3. Colony Formation Unit (CFU) Assay

One-five hundred cultured cells were suspended in 1.1 mL of MethoCult GF H4435 (STEMCELL Technologies, Vancouver, BC, Canada), plated in a 35 mm culture dishes, and incubated in 5% CO_2_ at 37 °C. Colonies were counted under the microscope on DAY12-14.

### 4.4. Reconstitution Analysis

Six-week old female Non-obese diabetic/Shi-scid/IL-2Rγnull (NOG) mice were provided by the CLEA Japan, Inc. (Shizuoka, Japan), and used as recipients of transplantation. Animals were handled according to the Guidelines for the Care and Use of Laboratory Animals of Kyushu University. To assess hematopoietic reconstitution, cultured cells were collected on DAY9 and the total progeny of CD34+ cells expanded in culture were transplanted into NOG recipient mice irradiated at 2.5 Gy. Sixteen weeks later, recipients were sacrificed by cervical dislocation and BM was harvested from femurs and tibias. Half of the BM cells from each recipient was then transplanted into irradiated NOG mice as secondary transplantation. The other half was analyzed for human-CD45 expression by flow cytometry. Three months after secondary transplantation, BM cells were harvested from recipients and analyzed for human-CD45 expression. This study was approved by Animal Care and Use Committee, Kyushu University.

### 4.5. siRNA Transfection

siRNA transfection was performed using a 4D-NucleofectorTM and a P3 Primary Cell 4D-NucleofectorTM X Kit (Lonza, Basel, Switzerland), following the manufacturer’s instruction. Briefly, 5 × 10^4^ UCB CD34+ cells were suspended in 20 μL Nucleofector solution containing supplements and 300 nM of siRNAs (Table S5) and electroporated using program E0-100. Cells were then resuspended in 180 μL pre-warmed culture medium and transferred into 48-well culture plates. Twenty-four hours later, some cells were collected to assess knockdown efficiency; three days after transfection, the rest were collected and used for a CFU assay.

### 4.6. RNA Extraction and Quantitative Reverse Transcription Polymerase Chain Reaction, RT-PCR (qRT-PCR)

RNA was extracted using an RNAqueous-Micro Kit (Ambion, Austin, TX, USA) following the manufacturer’s instruction. RNA was converted into cDNA using a High Capacity RNA-to-cDNA Kit (Ambion). qRT-PCR was performed with Fast SYBR^®^ Green Master Mix (Applied Biosystems, Foster City, CA, USA) and the StepOnePlus real-time PCR system (Applied Biosystems, Foster City, CA, USA). ACTB served as the reference gene. Primer sequences used were listed in Table S6.

### 4.7. Gene Expression Microarrays

The cRNA was amplified, labeled, and hybridized to a 60K Agilent 60-mer oligomicroarray (SurePrint G3 Human Gene Expression Microarray 8 × 60K v3) according to the manufacturer’s instructions. All hybridized microarray slides were scanned by an Agilent scanner. Relative hybridization intensities and background hybridization values were calculated using Agilent Feature Extraction Software (9.5.1.1).

Raw signal intensities and Flags for each probe were calculated from hybridization intensities (gProcessedSignal), and spot information (gIsSaturated, etc.), according to the procedures recommended by Agilent. (Flag criteria on GeneSpring Software. Absent (A): “Feature is not positive and significant” and “Feature is not above background”. Marginal (M): “Feature is not Uniform”, “Feature is Saturated”, and “Feature is a population outlier”. Present (P): others.) Additionally, the raw signal intensities of two samples were log2-transformed and normalized by quantile algorithm with ‘preprocessCore’ library package on Bioconductor software. We selected probes that call ‘P’ flag at least one sample, excluding lincRNA probes. To identify up or down-regulated genes, we calculated Z-scores and ratios (non-log scaled fold-change) from the normalized signal intensities of each probe for comparison between control and experiment sample. Then, we established criteria for regulated genes: (up-regulated genes) Z-score ≥ 2.0 and ratio ≥ 2-fold, (down-regulated genes) Z-score ≤ −2.0 and ratio ≤ 0.5. The regulated genes were analyzed through the use of ingenuity pathway analysis (IPA) software (Qiagen GmbH, Hilden, Germany).

### 4.8. Cell Proliferation Assay

Cell proliferation was determined by a Cell Proliferation BrdU colorimetric assay according to the manufacturer’s instructions (Roche, Mannheim, Germany). Briefly, PB CD34+ cells were treated with 10 µg/mL of SL-13R for 3 days. BrdU labeling solution was added and incubated for 2 h at 37 °C followed by fixation and DNA denaturation. BrdU-POD antibody was added and incubated for 90 min at room temperature. After washing, cells were analyzed by using BD FACSCanto flow cytometer with BD FACSDiva software (BD Bioscience, San Jose, CA, USA).

### 4.9. Intracellular Detection of SL-13R

UCB CD34+ cells were cultured in the presence of biotin-conjugated SL-13R. After culture, cells were collected and cytospin onto glass slides (Matsunami, Osaka, Japan). The cells cells were fixed with 1% paraformaldehyde (PFA)/PBS, permeabilized with 0.3% Triton X-100/PBS, blocked with 1% BSA/PBS, and stained with AlexaFluor 488- or Allophycocyanin (APC)-conjugated streptavidin (Life Technologies, Carlsbad, CA, USA). Nuclei were counterstained with TOTO-3 (Life Technologies, Carlsbad, CA, USA). Samples were assessed using a FluoView 1000 Confocal Microscope (Olympus, Tokyo, Japan) and FACSAria cell sorter (BD Biosciences, San Jose, CA, USA).

### 4.10. Immunoprecipitation and LC-MS/MS Analysis

UCB CD34+ cells were cultured in medium with or without biotin-conjugated SL-13R. After 12 hr, cells were collected and lysed with ice cold IP Lysis/Wash Buffer from the Pierce Crosslink IP Kit (Thermo Fisher Scientific, Waltham, MA, USA). Immunoprecipitation was performed following manufacturer’s instruction of Pierce Classic IP kit (Thermo Fisher Scientific Inc., Rockford, IL, USA). Briefly, 1 mg of protein was incubated with 2 μg of murine anti-biotin IgG2a (BioLegend, San Diego, CA, USA) at 4 °C overnight. To capture protein-peptide-antibody complex, protein A/G agarose beads were added and incubated at 4 °C for one hour. The beads were then washed three times with IP lysis/wash buffer, once with conditioning buffer and subjected to elution buffer pH 2.8. After neutralizing low pH with 1 M Tris pH 9.5, proteins were digested by trypsin and resulting peptides were subjected to liquid chromatography tandem mass spectrometry (LC-MS/MS) (Waters, Tokyo, Japan). After immunoprecipitation, LC-MS/MS analysis and datasets were analyzed using MASCOT Search software (Matrix Science, Tokyo, Japan).

### 4.11. Immunocytochemistry

UCB CD34+ cells were attached to glass slides. After thorough air-drying, cells were fixed with 1% paraformaldehyde (PFA)/PBS, permeabilized with 0.3% Triton X-100/PBS, and blocked with 1% BSA/PBS. The primary antibodies used are shown in Table S4. Nuclei were counterstained with TOTO-3. Images were obtained under a FluoView 1000 Confocal Microscope. 

### 4.12. Proximity Ligation Assay

To examine SL-13R-protein interaction, proximity link assay was performed. UCB CD34+ cells were cultured with biotin-conjugated SL-13R for 48 h. Cytospin, cell fixation and permeabilization are similar to previous mention in Immunocytochemistry. After overnight incubation with rabbit anti-AHNAK, ANXA2, and PLEC antibody (Table S4) and mouse anti-biotin antibody, Duolink In Situ PLA Probe anti-rabbit MINUS and anti-mouse PLUS were applied to samples (Sigma-Aldrich, Uppsala, Sweden). Proximally located antibody-binding probes were ligated with oligonucleotide by ligase at 37℃ for 30 min using Duolink In Situ Detection Reagent Green (Sigma-Aldrich, Uppsala, Sweden). Rolling Circle Amplification of probes was performed using DNA polymerase at 37℃ for 100 min. After wash with Buffer B, nuclei were stained with TOTO-3, and analysed using a FluoView 1000 Confocal Microscope.

### 4.13. Statistical Analysis

Datasets are represented as means ± standard deviation. For statistical analysis, Student t test or analysis of variance followed by Tukey’s post hoc test was carried out using GraphPad PRISM 6 software (GraphPad Software, Inc., La Jolla, CA, USA).

## Figures and Tables

**Table 1:** List of pathways regulated by SL-13R

Ingenuity Canonical Pathways	−log (*p*-Value)	Regulation	z Score	Ratio	Molecules
Role of NFAT in Regulation of the Immune Response	3.11	up	1	0.072	AKT3, CSNK1G1, CSNK1G2, GATA4, GNA13, GNAI3, GNAG2, GNG4, ITK, MAP2K1, ORAI1, PPP3R1, RAP1A
Role of BRCA1 in DNA Damage Response	2.37	up	0.45	0.088	ATM, ATR, BLM, FANCL, NBMN, PBRM1, UIMC1
Role of NFAT in Cardiac Hypertrophy	2.05	up	1.27	0.056	AKT3, CACNA1A, GATA4, GNAI3, GNG2, GNG4, HDAC11, MAP2K1, PPP3R1, PRKAR2B, RAP1A, TGFBR2
Superpathway of Methionine Degradation	1.85	up	1	0.11	CBS/CBSL, EEF1AKMT2, MAT2A, MCEE
Relaxin Signaling	1.85	up	1.34	0.06	AKT3, GNA13, GNAI3, GNG2, GNG4, MAP2K1, PDE6C, PRKAR2B, RAP1A
Protein Kinase A Signaling	1.84	up	1	0.045	ADD3, DUSP12, GNA13, GNAI3, GNG2, GNG4, GYS1, MAP2K1, PDE6C, PPP3R1, PRKAR2B, PTPN14, PTPRR, RAP1A, ROCK1, TCF3, TGFBR2, YWHAE
Ephrin Receptor Signaling	1.76	up	1	0.056	ADAM10, AKT3, GNA13, GNAI3, GNG2, GNG4, ITGA4, MAP2K1, RAP1A, ROCK1
Telomerase Signaling	1.71	up	0.45	0.065	AKT3, HDAC11, IL2RB, MAP2K1, PPP2R3A, RAP1A, TERF2IP
PI3K/AKT Signaling	1.7	up	1.41	0.061	AKT3, EIF4E, GYS1, ITGA4, MAP2K1, PPP2R3A, RAP1A, YWHAE
Cardiac Hypertrophy Signaling (Enhanced)	1.6	up	1.1	0.041	AKT3, ATP2A2, CACNA1A, EIF4E, GATA4, GNA13, GNAI3, GNG2, HDAC11, IL10RA, IL2RB, ITGA4, MAP2K1, PDE6C, PPP3R1, PRKAR2B, RAP1A, ROCK1, TGFBR2, TNFSF10
Insulin Receptor Signaling	1.58	up	0.71	0.058	AKT3, EIF4E, GYS1, MAP2K1, PRKAR2B, RAP1A, SOCS3, VAMP2
Leukocyte Extravasation Signaling	1.51	down	−1.27	0.051	BMX, F11R, FER, GNAI3, ITGA4, ITGAL, ITK, MMP24, RAP1A, ROCK1
P2Y Purigenic Receptor Signaling Pathway	1.36	up	1.63	0.055	AKT3, GNAI3, GNG2, GNG4, MAP2K1, PRKAR2B, RAP1A
Neurotrophin/TRK Signaling	1.35	up	1	0.066	MAP2K1, NTRK1, RAP1A, SPRY1, SPRY2
AMPK Signaling	1.34	up	1.63	0.047	AK4, AKT3, CHRNA3, EEF2K, GYS1, PBRM1, PFKFB3, PPP2R3A, PRKAR2B, RAB27A
IGF-1 Signaling	1.3	up	1.34	0.058	AKT3, MAP2K1, PRKAR2B, RAP1A, SOCS3, YWHAE

## Data Availability

The Gene Expression Microarrays data are available through the Gene Expression Omnibus, under accession numbers: GSE167599.

## Supplementary Materials

The following are available online. Figure S1: Ex vivo expansion of human UCB HSPCs by SL-13R; Figure S2: Ex vivo expansion of human PB HSPCs by SL-13R; Figure S3: SL-13R treatment increase incorporation of BrdU; Figure S4: Expression of candidate genes for SL-13R binding; Figure S5: Gene knockdown effects of siRNA for ERLN2, HIST4H4, HSPB1, and RAB30; Figure S6: Knockdown effects of ERLN2 and HIST4H4 on number of total CFU, Table S1: List of cell cycle related genes; Table S2: List of cell proliferation related genes; Table S3: List of peptides identified in the LC-MS/MS analysis; Table S4: List of antibodies for ICC and flow cytometry; Table S5: List of siRNA; Table S6:List of primers for qRT-PCR.

## Sample Availability

Samples of the compounds, SL-13R, are available from Science Lustre Ltd.
