# Supplementary material for: The Bioactive Peptide SL-13R Expands Human Umbilical Cord Blood Hematopoietic Stem and Progenitor Cells In Vitro
Source: Molecules. 2021 Apr 1;26(7):1995. doi: 10.3390/molecules26071995 (PMC8036704; doi:10.3390/molecules26071995)
Supplement: Supplementary file 1 [file molecules-26-01995-s001.pdf]

Table S1 List of cell cycle related genes.

Diseases or Functions Annotation	p-value	Activation z-score	Molecules
Senescence of cells	5.1E-05	-0.53	ADD3,AIMP2,AR,ATM,ATR,BLM,BTG3,DYRK1A,EIF4E,GATA4,GPATCH8,HMGA2,ID1,MAP2K1,MAPKAPK5,ME2,MITF,NBN,PBRM1,PPP1R13B,TAF1C,TCF3,TGFBR2,UIMC1,ZEB2
G2 phase	0.00025	-1.51	AR,ATM,ATR,BLM,CCNY,EEF2K,EIF2AK2,EP400,HAUS7,HMGA2,MAP2K1,MAPRE1,MXI1,NBN,OFD1,PRKAR2B,RBBP6,SPHK2,TAF6,TAOK2,TGFBR2,TTI1,UIMC1,YWHAE,ZEB2
Interphase	0.00034	-0.25	AFAP1L2,AR,ATM,ATR,BCAT1,BLM,BTG3,CCL3,CCNY,CDC6,DAB2,DYRK1A,EEF2K,EIF2AK2,EIF4E,EP400,FZR1,HAUS7,HMGA2,ID1,KIT,LBH,MAP2K1,MAPRE1,MAX,MCM2,MINDY3,MXI1,NBN,OFD1,PFKFB3,PLAG1,PPP1R26,PRKAR2B,RBBP6,RXRA,SPHK2,SPRY2,TAF6,TAOK2,TCF3,TFCP2,TGFBR2,TTI1,UIMC1,USP36,YWHAE,ZEB2
Replicative senescence of fibroblast cell lines	0.00042	-0.82	ADD3,ATM,GPATCH8,NBN,PBRM1,TAF1C
Senescence of fibroblast cell lines	0.00048	-0.67	ADD3,AIMP2,ATM,BTG3,DYRK1A,GATA4,GPATCH8,ID1,ME2,NBN,PBRM1,TAF1C,TCF3
Mitosis	0.00050	-0.54	AFAP1L2,AR,ATM,ATR,AZI2,BLM,BTG3,CDC6,ELOC,FER,FOXC1,GNA13,GNAI3,HAUS7,IL2RB,ITGAL,KIT,MAP2K1,MAX,MITF,NBN,NTRK1,PBRM1,PEBP1,PKN2,SASS6,SPHK2,SPRY2,TAF6,TGFBR2,THBS1,TUBB3,YWHAE
G2/M phase	0.000592	-1.70	AR,ATM,ATR,BLM,CCNY,EIF2AK2,EP400,HAUS7,HMGA2,MAPRE1,MXI1,NBN,OFD1,PRKAR2B,RBBP6,SPHK2,TAOK2,TGFBR2,TTI1,UIMC1,YWHAE
Cell cycle progression	0.00061	-1.20	AFAP1L2,AMER1,AR,ARHGAP32,ATM,ATR,AZI2,BLM,BTG3,CCL3,CDC6,EIF2AK2,EIF4E,ELOC,FER,FOXC1,FZR1,GAS7,GATA4,GNA13,GNAI3,HAUS7,ID1,IL2RB,IRF9,ITGAL,KIT,LBH,MAP2K1,MAX,MIR17HG,MITF,MXI1,NBN,NR4A3,NTRK1,PBRM1,PEBP1,PES1,PHC1,PHF6,PKN2,PLAG1,PPP1R13B,PPP2R3A,PRKAR2B,RARG,SASS6,SETD7,SOCS3,SPHK2,SPRY2,TAF6,TCF3,TFCP2,TGFBR2,THBS1,THRA,TNFSF10,TUBB3,UHRF2,YWHAE
Replicative senescence of cells	0.00083	-0.82	ADD3,ATM,ATR,GPATCH8,MAPKAPK5,NBN,PBRM1,TAF1C,UIMC1
Ploidy of cells	0.0033	-1.14	AKT3,ATM,DYRK2,IRAK4,MAP2K1,RGPD4,ROCK1,SPRY2,SUDS3,TCIRG1,TGFBR2
S phase checkpoint control	0.0034	-1.09	ATM,EIF4E,NBN,TTI1
G1 phase of fibroblast cell lines	0.0063	0	CDC6,DYRK1A,EP400,FZR1,MAP2K1,MCM2,TCF3
G1/S phase transition	0.0068	1.13	BCAT1,BTG3,CDC6,EIF2AK2,EIF4E,FZR1,KIT,MAP2K1,MAX,MCM2,PLAG1,PPP1R26,SPRY2,TCF3,TFCP2,ZEB2
Checkpoint control	0.0075	-1.67	ATM,ATR,CDC6,CSNK1G1,EIF4E,NBN,TTI1,UIMC1
Interphase of cancer cells	0.0076	-1	ATM,ATR,CDC6,TGFBR2
S phase	0.0085	0.26	AR,ATM,ATR,BLM,CCL3,CDC6,EP400,FZR1,ID1,LBH,MAP2K1,MAX,MXI1,NBN,PFKFB3,TGFBR2,TTI1
Modification of chromatin	0.0092	0.45	ATM,BAZ2B,GATA4,HDAC11,PBRM1,SUPT16H,TBX21,ZBTB7A
Ploidy	0.0093	-1.39	AKT3,ATM,DYRK2,EP400,IRAK4,MAP2K1,RGPD4,ROCK1,SPRY2,SUDS3,TCIRG1,TGFBR2

Table S2 List of cell proliferation related genes.

Diseases or Functions Annotation	p-value	Activation z-score	Molecules
Production of lymphocytes	0.00012	-0.87	ADA,IL2RB,ITGA4,KIT,SOCS3,TBX21,TCF3,THRA
Growth of yeast	0.0003	1.06	BLM,DHX15,DUSP12,EIF2AK2,MAP2K1,MAPRE1,PIGN,RCL1,RGPD4 (includes others),SLC25A14
Production of cells	0.00056	-1.11	ADA,ATM,CCL3,IL2RB,INHBB,ITGA4,KIT,MAP2K1,SOCS3,TBX21,TCF3,TGFBR2,THRA
Proliferation of connective tissue cells	0.00059	-1.18	AKT3,AR,ARHGAP32,ATM,ATR,BLM,BMX,CDC6,EIF2AK2,EMD,EP400,FNDC3B,FOXC1,GOLPH3,HMGA2,HUWE1,INHBB,LIMA1,MAP2K1,NAB1,NAB2,NBN,PFKFB3,PHF14,PRKAR2B,RARG,RXRA,SDC4,SETDB1,SOCS3,SP2,SPHK2,SPRY2,TACC1,TCIRG1,TGFBR2,THRA,TNFSF10,UIMC1
Production of T lymphocytes	0.00075	0.22	ADA,ITGA4,KIT,SOCS3,THRA
Cell proliferation of melanoma cell lines	0.00098	1.23	AKIRIN2,AKT3,ATM,EIF4E,GOLPH3,KIDINS220,KIT,MAP2K1,MITF,PEAK1,PEBP1,THBS1,TLR3,TNFSF10,ZEB2
Cell proliferation of tumor cell lines	0.0017	0.72	ACSL4,ADAM10,AKIRIN2,AKT3,AR,ARCN1,ASPH,ATM,ATP2A2,ATP6AP1,ATR,BMX,BTG3,CASZ1,CCL3,DAB2,DPF2,EED,EEF2K,EIF4E,ELOVL7,ENAH,FDXR,FOXC1,FZR1,GATA4,GNG4,GOLPH3,HMGA2,HOTAIRM1,HTATIP2,HUWE1,ID1,IL2RB,ILF3,KIDINS220,KIT,LIMA1,MAP2K1,MAPRE1,MAX,MCM2,MINDY3,MIR17HG,MITF,MXI1,NAB2,NR2C2,NTRK1,OSBPL5,PBRM1,PEAK1,PEBP1,PFKFB3,PHC1,PHF6,PLAG1,PPP1R13B,PRKAR2B,PRRC2C,PTPN14,PTPRR,RARG,RBBP6,RBM17,RFX1,RXRA,SDC4,SETD7,SETDB1,SLC36A1,SOCS3,SPHK2,SPRY2,SRSF5,SUDS3,SUFU,TAF6,TCF3,TCIRG1,TGFBR2,THBS1,THRA,TLR3,TNFSF10,TNIK,TRA2A,TUBB3,USP36,VAMP2,ZBTB7A,ZEB2,ZNF451,ZSCAN1
Formation of thymocytes	0.0017	-0.45	ADAM10,EED,IL2RB,ITGAL,ITK,KIT,MAP2K1,NAB1,RPL22,TCF3
Development of hematopoietic progenitor cells	0.008	-0.65	ADAM10,EED,EIF2AK2,HIST1H4C,HMGA2,IL2RB,ITGAL,ITK,KIT,MAP2K1,NAB1,NR4A3,RPL22,TCF3,THBS1,TNRC6A,ZEB2,ZFPM1
Proliferation of prostate cell lines	0.0085	-0.22	AR,ATP2A2,DAB2,TGFBR2
Proliferation of neuroblastoma cell lines	0.0092	-0.40	CASZ1,FZR1,HMGA2,MXI1,NTRK1,RARG,RFX1,TNFSF10
T cell development	0.0094	-1.85	ADA,ADAM10,ATM,BLM,CCL3,EED,GATA4,ID1,IL10RA,IL2RB,ITGA4,ITGAL,ITK,KIT,MAP2K1,MIR17HG,NAB1,NBN,ORAI1,PKNOX1,POLM,RPL22,SOCS3,TBX21,TCF3,TCIRG1,TGFBR2,THRA,TLR3,TNFSF10,ZFPM1

Table S3 List of peptides identified in the LC-MS/MS analysis.

Name	Score
Annexin A2 isoform 1	248
Triosephosphate isomerase 1	245
Alpha-enolase	194
Neuroblast differentiation-associated protein AHNAK	147
Thioredoxin	121
Isoform A of Lamin-A/C	114
Calpain-1 catalytic subunit	112
Gamma-glutamylcyclotransferase	104
Cathepsin D	97
Glutathione S-transferase P	96
Heat-shock protein beta-1	86
Histone H4	77
Collagen alpha-2(1) chain	73
Isoform 1 of Plectin-1	68
Endoplasmic reticulum lipid raft-associated protein 2	65
Alpha-2-glycoprotein 1	62
Heat-shock protein 90Bb	62
L-lactate dehydrogenase C chain	61
Transaldolase	56
Ras-related protein Rab-30	54

Table S3

Table S4 List of antibodies for ICC and flow cytometry.

Target	Company	Catalog number
AHNAK	sigma	HPA019070
ANXA2	CST	8235
ERLN2	abcam	ab129207
HIST4H4	CST	13919
HSPB1	CAT	50353
PLEC	abcam	ab83497
RAB30	CUSABIO	CSB-PA613602LA01HU
CD34	eBioscience	12-0349-42
CD38	BioLegend	356608
CD45RA	BioLegend	304128
CD90	BD Biosciences	555595
CD49f	BioLegend	313616
CD45	BD Biosciences	555483

Table S5 List of siRNA.

Target		Mean of Knock down effect (%)
**AHNAK**	Hs_AHNAK_2903 Mission siRNA	37.5
**ANXA2**	Hs_ANXA2_7107 Mission siRNA	61.2
**ERLN2**	Hs_ERLIN2_7582 Mission siRNA	21.6
**HIST4T**	Hs_HIST4H4_4969 Mission siRNA	35.1
**PLEC**	Hs_PLEC1_2666 Mission siRNA	30.5

Table S6 List of primers for qRT-PCR.

Target		Primer sequence(5'-3')
ACTB	sense	catgtacgttgctatccaggc
	anti-sense	ctccttaatgtcacgcacgat
AHNAK	sense	tgaagtcttcagctcctgcag
	anti-sense	ttccactccatcttccgacttc
ANXA2	sense	gacttccgcaagacgatggt
	anti-sense	tggtatcccgagcatcttggt
CAPN1	sense	caggcaacaacctcaacaagaa
	anti-sense	gcagcaaacgaaaaagtcaaagt
COL1A2	sense	ccaactaagcctctcagaacatca
	anti-sense	ccctctacaatgacagcctttttc
CTSD	sense	gctggatggaccacaactacaa
	anti-sense	gcgtgcccgagccatag
ENO1	sense	tgctgctgctgaagactcgtatt
	anti-sense	ctcgcagcccgctacct
ERLIN2	sense	cattcaaggtgtgcgggtaa
	anti-sense	ggcggcaatgagaaggttt
GGCT	sense	agagaggaaggacctgcgaaa
	anti-sense	acttgttttgccttgggatttg
GSTP1	sense	ggcacttgaagccttttgaga
	anti-sense	caggttgtagtcagcgaaggagat
HIST4H4	sense	aacgccatttgtggtctcatc
	anti-sense	ttggcgtgctccgtgtaag
HSP90AB1	sense	attcgcatgaccaatcctgatc
	anti-sense	agtgcccaatgatggagatgtc
HSPB1	sense	gcgtgtcccaggatgtcaac
	anti-sense	gtgtatttcggcgtgaagca
LDHC	sense	tcttaggagagtccacccagttt
	anti-sense	accattccgccccaagac
LMNA	sense	ccgcaagacccaagactca
	anti-sense	ttggtattgggcgctttca
PLEC	sense	caacctgctgaactcctccaa
	anti-sense	ccatctcattcagccacattagc
RAB30	sense	aatccttccgttgccttcct
	anti-sense	ctcctcaatcttgttgcccacta
TALDO1	sense	aatcgtatgagcccctggaaga
	anti-sense	gaggcggcgatgacaatg
TP1	sense	tgtgtcggccattggtactc
	anti-sense	cgcatcagagaccttggactt
TXN	sense	cacatcctgagagtcatccacatt
	anti-sense	gggccttgcagaatgatcaa

Table S6

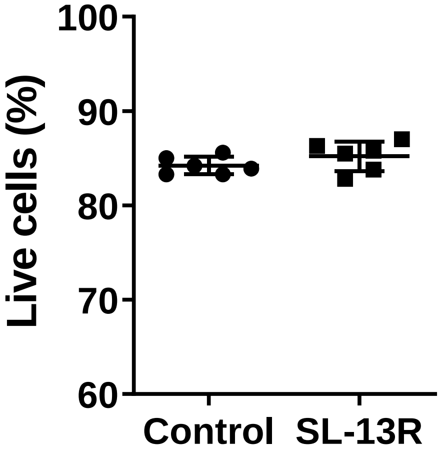

Fig. S1. Ex vivo expansion of human PB HSPCs by SL-13R
UCB CD34$^+$ cells were cultured with or without SL-13R for 9 days and analyzed percentage of live cells. . n=6; 6 donor.

Figure S1

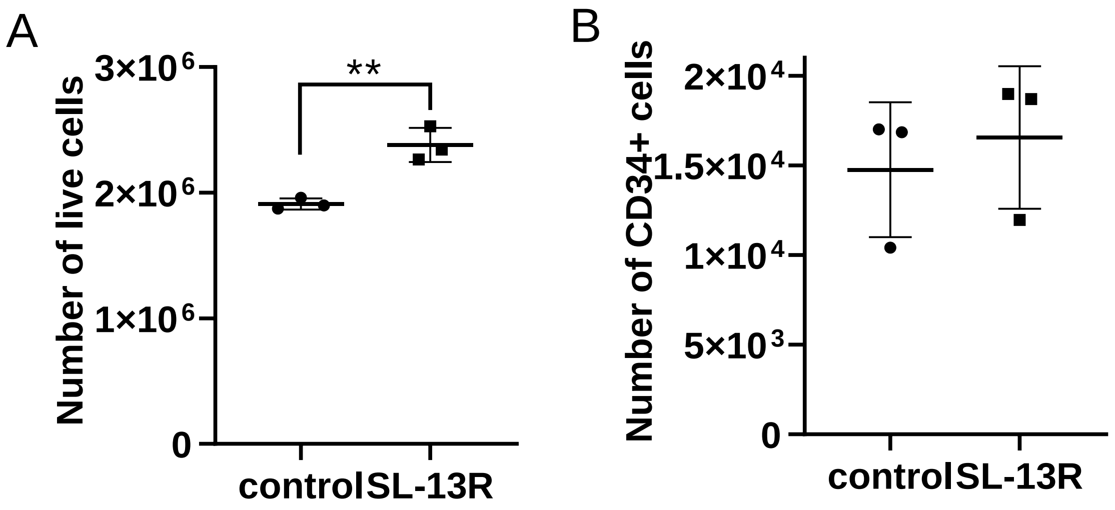

Fig. S2. Ex vivo expansion of human PB HSPCs by SL-13R

Human PB $CD34^{+}$ cells were cultured with or without SL-13R for 9 days. n=3; 3 donor (A) Number of live cells. (B) Number of CD34+ cells. The control was PBS treatment. Student t test was used to test intergroup differences. **$p < 0.01$.

Figure S2

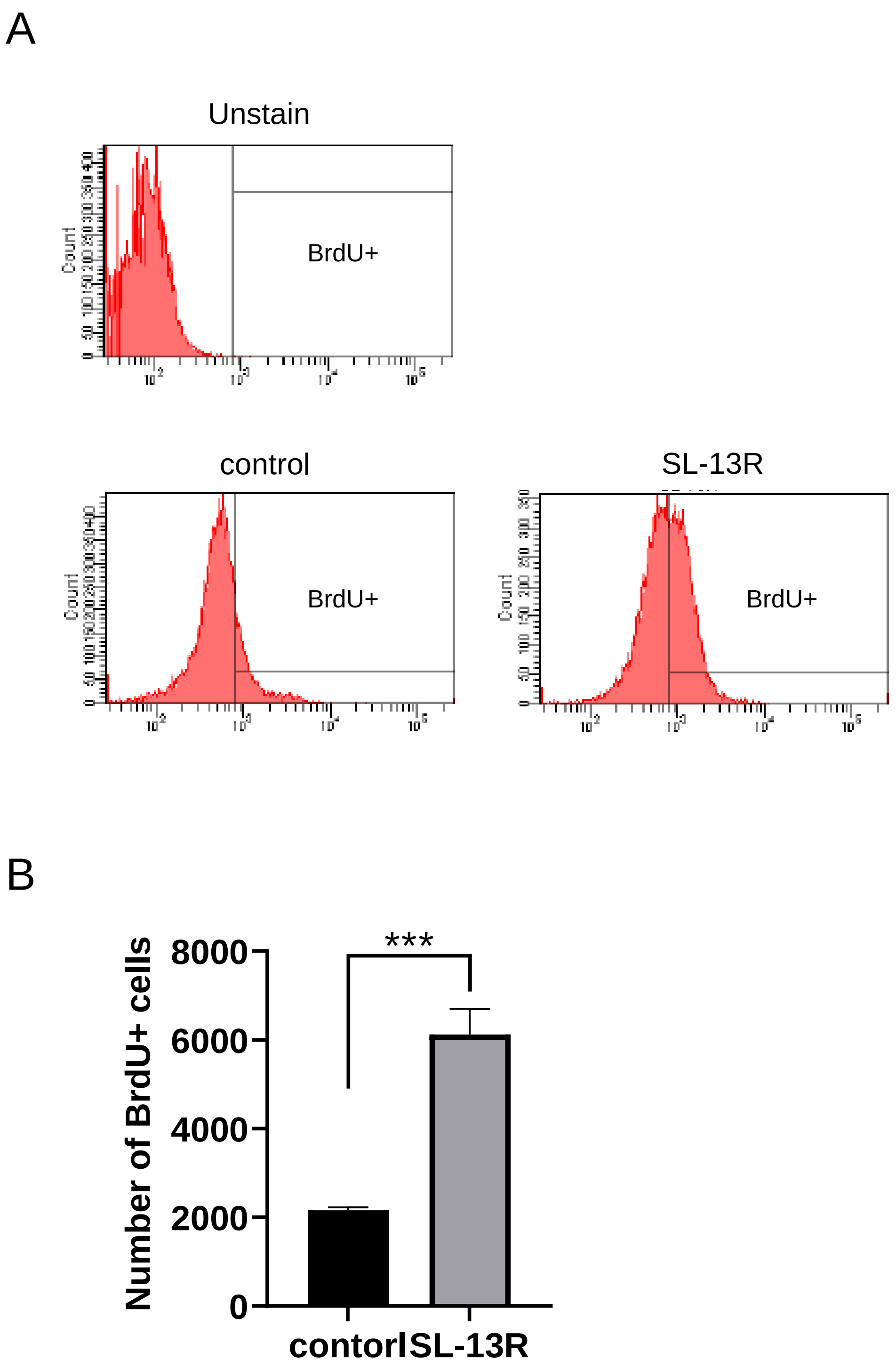

Fig. S3. SL-13R treatment increase incorporation of BrdU
Human PB $CD34^+$ cells were cultured with or without SL-13R and added BrdU on day 3 and analyzed BrdU positive cells. (A) Histogram of BrdU positive cells cultured with or without SL-13R, (B) Number of BrdU positive cells cultured with or without SL-13R for 3 days. Student t test was used to test intergroup differences. n=3 ***$p < 0.005$.

Figure S3

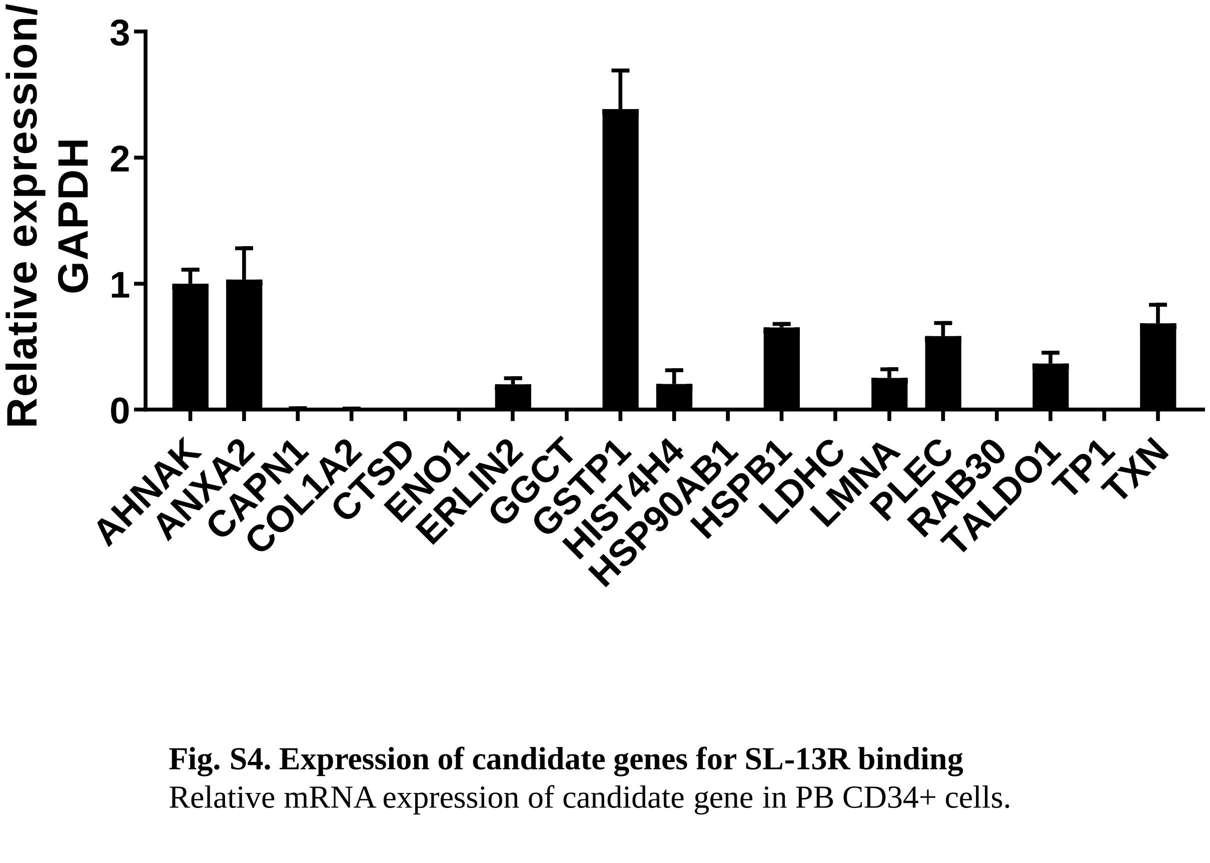

Fig. S4. Expression of candidate genes for SL-13R binding
Relative mRNA expression of candidate gene in PB CD34+ cells.

Figure S4

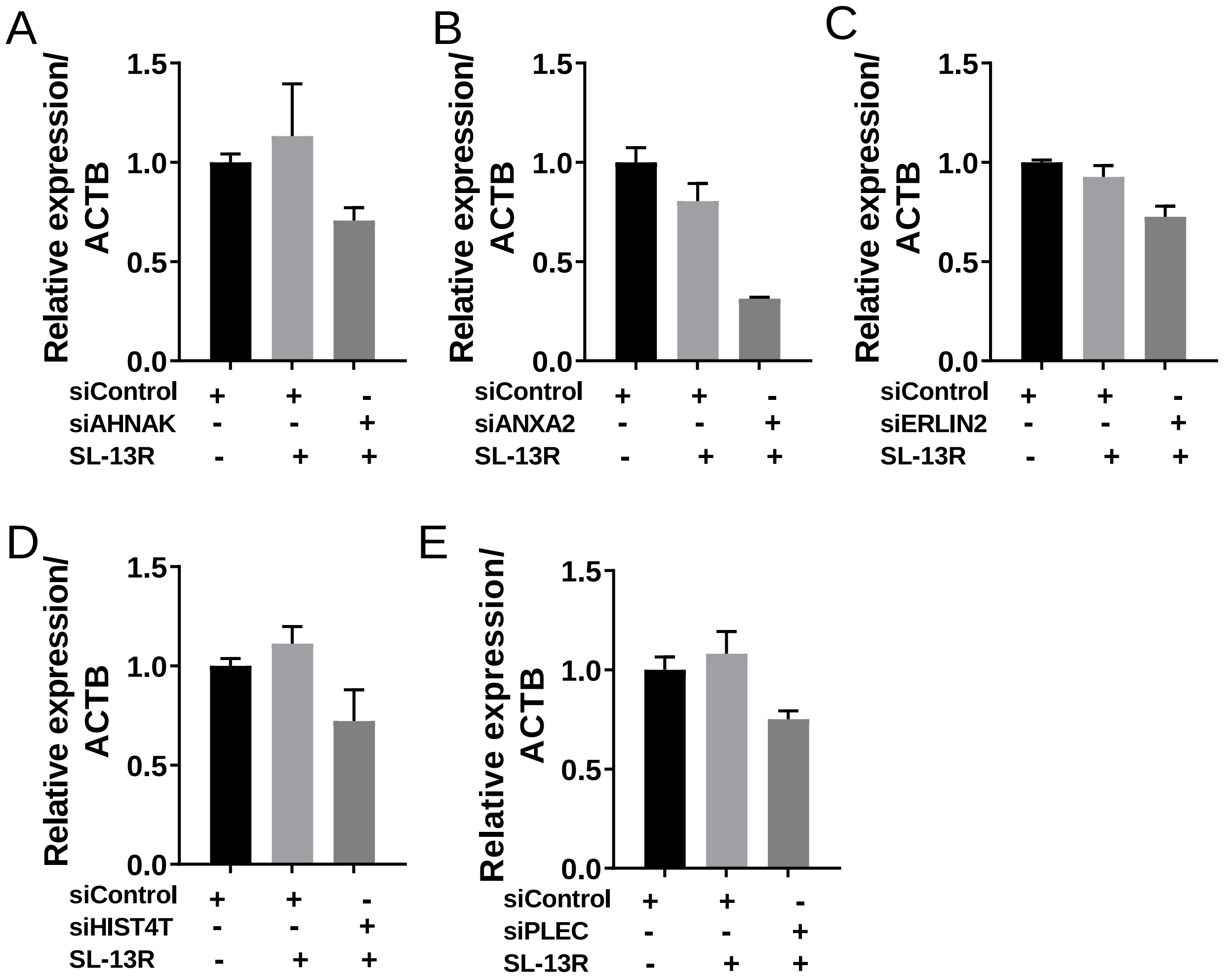

Fig. S5. Gene knockdown effects of siRNA for ERLN2, HIST4H4, HSPB1, and RAB30
Human UCB CD34^{+} cells were cultured with or without SL-13R and electroporation of siRNA for ERLN2, HIST4H4, HSPB1, and RAB30 for 24 kr and analyzed gene expression by qRT-PCR. (A) siRNA for AHNAK, (B) siRNA for ANXA2, (C) siRNA for ERLN2, (D) siRNA for HIST4T, (E) siRNA for PLEC.

Figure S5

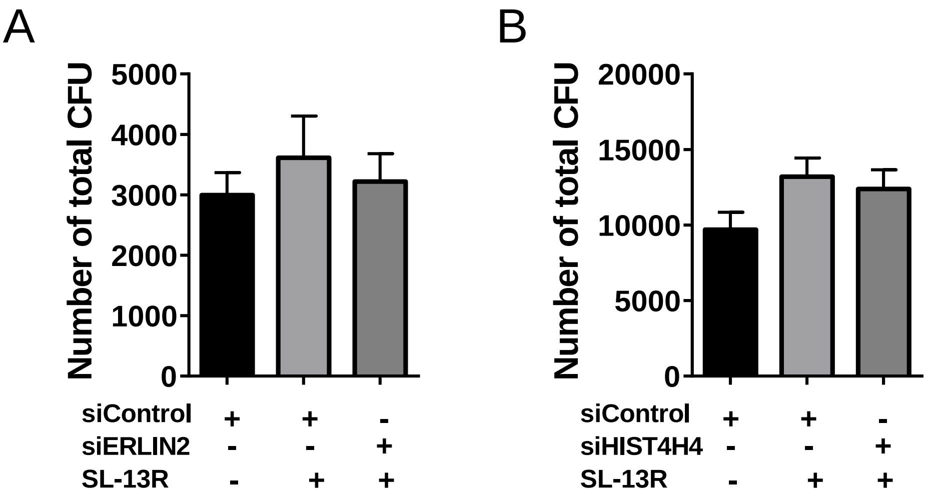

Fig. S6. Knockdown effects of ERLN2 and HIST4H4 on number of total CFU
Human UCB CD34$^+$ cells were cultured with or without SL-13R and electroporation of siRNA for ERLN2 or HIST4H4 for 3 days and performed CFU assay. n=3 (A) siRNA for ERLN2, (B) siRNA for HIST4H4.

Figure S6
